# The neutrophil-mobilizing cytokine interleukin-26 in the airways of long-term tobacco smokers

**DOI:** 10.1042/CS20180057

**Published:** 2018-05-21

**Authors:** Karlhans Fru Che, Ellen Tufvesson, Sara Tengvall, Elisa Lappi-Blanco, Riitta Kaarteenaho, Bettina Levänen, Marie Ekberg, Annelie Brauner, Åsa M. Wheelock, Leif Bjermer, C. Magnus Sköld, Anders Lindén

**Affiliations:** 1 Unit for Lung and Airway Research, Institute of Environmental Medicine, Karolinska Institutet, Stockholm SE-171 77, Sweden; 2 Respiratory Medicine and Allergology, Department of Clinical Sciences, Lund University, Lund, Sweden; 3 Department of Internal Medicine and Clinical Nutrition, Institute of Medicine, Sahlgrenska Academy at the University of Gothenburg, Gothenburg, SE-405 30, Sweden; 4 Department of Pathology, Center for Cancer Research and Translational Medicine, Oulu University Hospital and University of Oulu, Oulu, Finland; 5 Respiratory Medicine, Research Unit of Internal Medicine, Medical Research Center, University of Oulu and Oulu University Hospital, Oulu, Finland; 6 Department of Microbiology, Tumor and Cell Biology, Division of Clinical Microbiology, Karolinska University Hospital and Karolinska Institutet, Stockholm SE-171 76, Sweden; 7 Respiratory Medicine Unit, Department of Medicine Solna and Center for Molecular Medicine, Karolinska Institutet, Stockholm SE-171 76, Sweden; 8 Lung Allergy Clinic, Karolinska University Hospital, New Karolinska Solna, Stockholm SE-171 76, Sweden

**Keywords:** Airways, host defense, IL-26, inflammation, Smokers with COPD, Th17 cytokines

## Abstract

Long-term tobacco smokers with chronic obstructive pulmonary disease (COPD) or chronic bronchitis display an excessive accumulation of neutrophils in the airways; an inflammation that responds poorly to established therapy. Thus, there is a need to identify new molecular targets for the development of effective therapy. Here, we hypothesized that the neutrophil-mobilizing cytokine interleukin (IL)-26 (IL-26) is involved in airway inflammation amongst long-term tobacco smokers with or without COPD, chronic bronchitis or colonization by pathogenic bacteria. By analyzing bronchoalveolar lavage (BAL), bronchail wash (BW) and induced sputum (IS) samples, we found increased extracellular IL-26 protein in the airways of long-term smokers *in vivo* without further increase amongst those with clinically stable COPD. In human alveolar macrophages (AM) *in vitro*, the exposure to water-soluble tobacco smoke components (WTC) enhanced *IL-26* gene and protein. In this cell model, the same exposure increased gene expression of the IL-26 receptor complex (*IL10R2 and IL20R1*) and *nuclear factor κ B (NF-κB);* a proven regulator of IL-26 production. In the same cell model, recombinant human IL-26 *in vitro* caused a concentration-dependent increase in the gene expression of *NF-κB* and several pro-inflammatory cytokines. In the long-term smokers, we also observed that extracellular IL-26 protein in BAL samples correlates with measures of lung function, tobacco load, and several markers of neutrophil accumulation. Extracellular IL-26 was further increased in long-term smokers with exacerbations of COPD (IS samples), with chronic bronchitis (BAL samples ) or with colonization by pathogenic bacteria (IS and BW samples). Thus, IL-26 in the airways emerges as a promising target for improving the understanding of the pathogenic mechanisms behind several pulmonary morbidities in long-term tobacco smokers.

## Introduction

Chronic obstructive pulmonary disease (COPD) will soon constitute the third most common disease-related cause of mortality in the world. Of note, long-term tobacco smoking remains the leading cause of COPD in the industrialized world and an important one for its common comorbidity chronic bronchitis [1,2]. Amongst long-term smokers, with or without pulmonary morbidities such as COPD or chronic bronchitis, bacterial infections are common and this is true even amongst former smokers [2–5]. These bacterial infections trigger sustained exacerbations of disease in long-term smokers with COPD or chronic bronchitis, thereby contributing to decline in lung function and enhanced morbidity and mortality [3,5]. During these exacerbations, the local inflammation in the airways may become more severe but there is poor understanding of how this inflammation influences pulmonary morbidities in long-term smokers.

A growing body of evidence demonstrates that the local inflammation is characterized by an excessive accumulation of neutrophils in the airways of long-term tobacco smokers with or without pulmonary morbidities; a type of inflammation that in general responds poorly to therapy including glucocorticoids [2,4,6]. The referred, excessive accumulation of neutrophils occurs in parallel with bacterial colonization and an increased susceptibility to local infections. Of note, this accumulation of neutrophils can be observed when there are no detectable bacteria present, suggesting an inherent neutrophil activity, possibly due to a malfunction in the endogenous control of innate immunity [5].

Indeed, the endogenous control of innate immunity in human lungs is complex and displays signs of mechanistic redundancy, presumably due to its fundamental importance for the survival of the host [7]. Along these lines, the cytokine interleukin (IL)-26 (IL-26) constitutes yet another example of a cytokine that is involved in the immune response to bacterial endotoxin in the airways of healthy human subjects [8–10]. This cytokine is produced by human alveolar macrophages (AM) as well as Th17 cells [8–10]. Of mechanistic importance, IL-26 enhances the chemotactic response of human neutrophils to a bacterial and an inflammatory stimulus, thereby suggesting an important action exerted in a critical immune barrier [8–10]. Furthermore, IL-26 enhances the endotoxin-induced mobilization of neutrophils toward the bronchoalveolar space in an animal model *in vivo*, in a mouse strain that expresses the IL-26 receptor complex (IL-10R2 and IL20R1) [11]. Finally, IL-26 can be produced by human bronchial epithelial cells, thereby suggesting an important action exerted in yet another critical immune barrier [12]. In addition, there is a recently published experimental study suggesting that IL-26 acts as an antimicrobial peptide in the airways *in vivo*, one that directly kills extracellular bacteria through the formation of pores in their cell membrane[13].

There is now evidence that IL-26 is involved in Crohn’s disease [14], rheumatoid arthritis [15] and chronic hepatitis C infection [16], chronic inflammatory disorders that are characterized by an excessive local accumulation of neutrophils, resembling that observed in long-term tobacco smokers. Here, we reasoned that we can learn more about the pathogenic mechanisms behind pulmonary morbidities in long-term tobacco smokers by characterizing the involvement of IL-26 in the local inflammation of the airways of these subjects.

For the present study, we hypothesized that the neutrophil-mobilizing cytokine IL-26 is involved in the local inflammation amongst long-term tobacco smokers, with or without pulmonary morbidities. To address this hypothesis, we quantitated mRNA and protein for IL-26 in airway samples from long-term smokers, with or without pulmonary morbidities, in relation to local growth of pathogenic bacteria and to markers of neutrophil accumulation in the airways, lung function and to current and historic tobacco loads. We also characterized the effect of short-term exposure to tobacco smoke on local IL-26 concentrations in occasional smokers and that of water-soluble tobacco smoke components (WTC) on the production of IL-26 in human AM *in vitro.*

## Materials and methods

### Human cohorts

In the present study, samples and data from four established human cohorts (the ‘COSMIC’, ‘CYREBAC’, ‘BALO’, and ‘Smoke Expo’ cohorts) were analyzed, reported, and discussed. The principal clinical characteristics (demographics) of these cohorts are presented in Tables 1-3 and Supplementary Tables S1–S4. More information on the original setup, number of subjects recruited or excluded at the time each of these cohort materials were recruited can be found in previous relevant publications cited in this manuscript [17–22]. The examined subjects may thus have contributed with some data on certain basic clinical parameters in the referred publications, although grouped in a different manner, but no previous data on IL-26 has been published from these cohorts.

**Table 1:** Demographics for the subjects donating bronchoalveolar lavage samples (COSMIC cohort)

	Healthy nonsmokers	Smokers without COPD	Smokers with COPD
Subjects (*n*)	37	40	33
Sex (female/male)	18/19	20/20	16/17
Age (years)	60 (45–65)	52.5 (44–65)	61 (47–66)
FEV_1_% Pred	119 (89–147)	108.8 (91–140)	80 (51–97)
FEV_1_/FVC (%)	81 (70–93)	78 (71–88)	62 (43–75)
Smoking history	0	33.5 (15–66)	38 (17–62)
Current smokers	0	40	23/33
Ci/day for the last 6 months	0	17 (10–40)	15 (0–25)
CB (no/yes)	0	30/10	25/8
ICS	0	0	0
β antagonists	0	0	2
Anticholinergic	0	0	2

Data shown represent median and range. Abbreviations: CB, chronic bronchitis; Ci, cigarette; FEV_1_, forced expiratory volume in 1 s; FVC, forced vital capacity; ICS, inhaled corticosteroid.

#### The COSMIC cohort

This cohort (www.clinicaltrials.gov/ct2/show/NCT02627872) was gathered as previously described [17–21] and constituted our main cohort for the current study. The rationale for utilizing this cohort was to characterize IL-26 (protein or gene) in the small airways (bronchoalveolar lavage (BAL)) and the large airways (bronchial wash (BW)), as well in the airway mucosal (bronchial tissue biopsy of smokers with or without COPD), in relation to cellular sources, intracellular signaling molecules, tobacco load, growth of pathogenic bacteria, lung function, or chronic bronchitis. The cohort consists of age-matched healthy nonsmokers (*n*=37 for BAL samples and *n*=34 for BW samples), smokers without COPD with normal lung function (*n*=40 for BAL samples and *n*=33 for BW samples), and smokers with COPD (ever-smokers, GOLD 1-II/A-B) (*n*=33 for BAL samples and *n*=33 for BW samples) in both the genders. Notably, the smokers with COPD were in a clinically stable condition during sampling (BAL, BW, and bronchial tissue biopsy samples). Treatment with long-acting β agonists (LABA) and short-acting β agonists (SABA) as well as long-acting muscarinic antagonists (LAMA) and short-acting muscarinic antagonists (SAMA) were accepted. Treatment with any kind of glucocorticoids was not accepted (see Tables 1 and 2 for details).

**Table 2:** Demographics for the subjects donating BW samples (COSMIC cohort)

	Healthy nonsmokers	Smokers without COPD	Smokers with COPD
Subjects (*n*)	34	33	33
Sex (female/male)	17/17	16/17	16/17
Age (years)	60 (46–65)	53 (44–65)	62 (47–66)
FEV_1_% Pred	120 (92–141)	106 (91–130)	81.5 (51–97)
FEV_1_/FVC (%)	82 (71–91)	78 (71–88)	62.5 (43–69)
Smoking history	0	34 (15–84)	40 (17–51)
Current smokers	0	33	22
Ci/day for the last 6 months	0	17 (10–40)	11 (0– 25)
CB (no/yes)	0	24/9	25/8
ICS	0	0	0
β antagonists	0	0	3
Anticholinergic	0	0	2

Data shown represent median and range. Abbreviations: CB, chronic bronchitis; Ci, cigarette; FEV_1_, forced expiratory volume in 1 s; FVC, forced vital capacity; ICS, inhaled corticosteroid.

The smokers were matched in terms of smoking history and current smoking status (>10 cigarettes/day within the last 6 months), as confirmed by exhaled CO (See Tables 1 and 2 for demographics, for BAL and BW samples, respectively). All subjects underwent a clinical examination, chest X-ray, and spirometry (Jaeger Masterscope PC, CareFusion, Hong Kong, China). Individuals with a history of allergy or asthma were excluded from the study. *In vitro* screenings for the presence of specific IgE antibodies (Phadiatop, Pharmacia, Uppsala, Sweden) were negative. A history of infection or symptom worsening (exacerbation) during the last 3 months constituted exclusion criteria. Flexible bronchoscopy (Olympus Optical Co. Ltd,) was performed as previously described [23,24] according to standard clinical protocol. The instrument was passed nasally and a BW (10 ml × 1) was performed in right upper lobe segmental bronchus, followed by BAL (50 ml × 5) in middle lobe bronchus, utilizing sterile PBS solution (37°C) [24]. The instilled PBS solution was aspirated and collected in a siliconized plastic bottle kept on ice until it reached the lab. The bronchial tissue biopsies were collected by the use of pulmonary biopsy forceps with smooth edge jaws (Radial Edge® Biopsy Forceps, Boston Scientific, Boston, MA) [21,23,25]. Notably, chronic bronchitis was regarded as a distinct disease entity and the diagnostic criterion was based upon daily phlegm production (productive cough) during at least three consecutive months for at least 2 years [26].

#### The BALO cohort

This cohort was gathered as previously described [22]. The rationale for utilizing this cohort was to characterize IL-26 in the airways of smokers with COPD during stable clinical conditions and during exacerbations. Given that repeated sampling of the patients over time was required, these aspects had to be investigated in induced sputum (IS) samples. We thus investigated IL-26 protein concentrations in IS in relation to tobacco load, growth of pathogenic bacteria, lung function, and markers of neutrophil accumulation. In brief, the smokers with COPD (‘ever-smokers’ (*n*=33) were included following the GOLD stage standards. Notably, treatment with LABA and SABA as well as LAMA and SAMA were accepted. Treatment with inhaled corticosteroids (ICS) but not oral steroids was also accepted (see Table 3 for details). The IS samples from healthy individuals were also investigated (*n*=8). The patients completed two self-administered questionnaires; the Clinical COPD Questionnaire (CCQ) and Medical Research Council (MRC) dyspnea scale. All subjects underwent a physical and clinical examination, dynamic spirometry (Jaeger MasterScreen, Erich Jaeger GmbH, Würzburg, Germany), followed by impulse oscillometry (IOS) using a Jaeger MasterScreen Impulse Oscillometry System (Jaeger) and the reference values stipulated by Crapo et al. [27] were used. The COPD patients had at least one exacerbation for which antibiotics were needed in the preceding year and the study participants could not have a history of lung cancer or asthma, no exacerbation or respiratory infection within the preceding 6 weeks, and no use of exacerbation treatment for >3 weeks. Participants were included and spirometry done while they were in their stable state and were followed up monthly until they experienced an exacerbation or a minimum of 6 months had passed. The patients agreed to come for an extra visit (prior to scheduled visits) when exacerbating. Each parameter value of stable phase was calculated as an average value of all visits during stable phase for each individual and the same was true for the exacerbation phase. During all visits, patients inhaled 5 mg salbutamol and 0.5 mg ipratropium via nebuliser prior to all investigations. Sputum was induced by inhalation of nebulized 0.9 and 4.5% NaCl and the IS was divided into two aliquots. Sputum was also induced from young healthy nonsmokers as controls (see Table 3 for demographics).

**Table 3:** Demographics for the subjects donating IS samples (BALO cohort)

	Healthy nonsmokers	Smokers with COPD
Subjects (*n*)	8	33
Sex (female/male)	5/3	17/16
Age (years)	23 (21–39)	67 (48–81)
FEV_1_% Pred	98 (72–116)	46.7 (17–81)
FEV_1_/FVC (%)	83 (71–93)	46 (32–75)
Smoking history	0	40 (15–165)
Ci/day for the last 6 months	0	7 (2–30)
ICS	0	33
β antagonists	0	33
Anticholinergic	0	31

Data shown represent median and range. Abbreviations: Ci, cigarette; FEV_1_, forced expiratory volume in 1 s; FVC, forced vital capacity.

#### The Smoke Expo cohort

The rationale for utilizing this cohort [25] was to characterize the effects of short-term exposure of healthy occasional smokers to tobacco smoke on IL-26 protein concentrations in BAL samples primarily. Two groups of subjects with normal lung function (by spirometry) were recruited. Occasional smokers without COPD (*n*=5) and never-smokers without COPD (*n*=9), (see Supplementary Table S4 for demographics). All the subjects (the occasional smokers and never-smokers) had refrained from smoking and had no respiratory infections for ≥4 weeks prior to participating in the study. All subjects underwent two bronchoscopies, days 1 and 14, and BAL samples were collected during both the procedures. On days 12–13, the occasional smokers smoked, in total, ten filtered cigarettes of a commercial brand (tar: 10 mg, nicotine: 0.8 mg). The smoking status for each subject was controlled by measuring the urine cotinine level at the time of each of the two bronchoscopies. In order to be included, all subjects had to display cotinine levels <100 ng.ml^−1^ prior to bronchoscopy 1 and for the inclusion of occasional smokers at the time of bronchoscopy 2, these subjects had to display cotinine levels at least five-fold to those obtained at bronchoscopy 1. The BAL (50 ml × 3) was performed as previously described [25] utilizing PBS. The fluid was collected in a polypropylene tube and kept on ice until it reached the lab.

#### The CYREBAC cohort

The rationale for utilizing this cohort [8] was to characterize the role of WTC on the induction of *IL-26* gene and protein in AM *in vitro.* Here, BAL samples were harvested during bronchoscopy using a protocol that has been previously described [8], similar to that used in the ‘COSMIC cohort’ above.

#### Ethics statements

The COSMIC cohort (smokers with or without COPD and healthy nonsmoking controls, diary number 2006/959-31/1) [17,23] and the CYREBAC cohort (healthy nonsmokers, diary number 2012/1571-3) [8] were reviewed and approved by the regional committee for ethical review in Stockholm. The BALO cohort (smokers with COPD and healthy nonsmokers, diary number 2005/639) [22,27] and the Smoke Expo cohort (occasional and never-smokers without COPD, diary number S 313-00; T186-02) [25] were approved by the respective regional committee for ethical review in Lund and Gothenburg, Sweden. The referred cohorts were conducted in accordance with the declaration of Helsinki, after oral and written informed consents.

### Laboratory investigations on airway samples

#### Processing of samples

The BAL samples from ‘COSMIC cohort’, ‘Smoke Expo cohort’, and ‘CYREBAC cohort’) as well as BW (‘COSMIC cohort’) were filtered through a Dacron net (Millipore®) and centrifuged (400×***g*** for 10 min at 4°C) and the total cell counts and Trypan Blue exclusion were carried out. The cell-free BAL or BW samples were immediately frozen (−80°C) for subsequent analyses. We also prepared BAL cell cytospin slides for differential counts (stained with May–Grünwald and Giemsa) and for immunocytofluorescence (ICF) staining. Aliquoted cells were stored for further analysis. The bronchial tissue biopsies (COSMIC cohort) were immediately formalin-fixed and embedded in paraffin. The material was evaluated, and one representative tissue block from each case was selected for immunohistochemistry (IHC) analysis. For the IS from the BALO cohort, one aliquot from the COPD patients was processed in 0.65 mM DTT, filtered, and stored at –80°C for subsequent analyses.

#### Measurement of IL-26 protein concentrations in BAL, BW, IS samples and conditioned media

IL-26 protein concentrations were quantitated in cell-free BAL, BW, and IS samples in accordance with the manufacturer’s instructions (Cusabio Biotech®). [8] In brief, diluted samples and standards were added in duplicates to plates and incubated (2 h) at 37°C in 5% CO_2._ Biotin-conjugated detection antibody was then added (1 h) followed by the avidin-conjugated horseradish peroxidase (1 h). Plates were developed with tetramethylbenzidine (TMB) substrate (90 µl) and stopped with a stop solution (50 µl). The optical density was measured (450 nm) using a microplate reader (Model Spectra Max 250, Molecular Devices™, Sunnyvale, CA). The limit of detection for IL-26 protein was zero.

#### Immunostaining of IL-26 in BAL cells and bronchial tissue biopsies

The detection of IL-26 in BAL cells was by ICF staining as previously described [8] and in the bronchial tissue biopsies by IHC.

##### ICF

Air-dried frozen BAL cytospin slides were selected from eight randomized subjects (four men and four women) per group (healthy nonsmokers, smokers without COPD, and smokers with COPD) and stained as previously described [8]. The staining was assessed by using Zeiss LSM700 microscope (Zeiss, Oberkochen, Germany) and high-resolution images of each specimen were captured at 10×, 25×, or 63× magnification. Immunocytochemical expression was then analyzed by semiquantitative digitalized image analysis using ImageJ, NIH®. Briefly, color images were first converted into 8-bit image in gray scale. The polygon selection mode was used to outline the cells and then the area of positive immunostaining was estimated by the number of black pixels within measuring mode. Thus, the area fraction of positive staining was determined as the percentage of black pixels’ area. Data are presented as corrected total cell fluorescence (CTCF) = integrated density − (area of selected cell by the mean fluorescence of background readings).

##### IHC

Here, bronchial tissue biopsy specimens were selected from eight randomized subjects (four men and four women) per group (healthy nonsmokers, smokers without COPD, and smokers with COPD). The bronchial tissue biopsies were then processed and stained as previously described [28] using the primary monoclonal mouse anti-human IL-26 (Clone 197505, R&D MAB1375 antibody or mouse IgG2b isotype control (Clone 133303, R&D) accordingly [8]. In brief, tissue blocks were formalin-fixed, paraffin embedded, de-paraffinized, and rehydrated through graded alcohols. Antigen retrieval was performed with Tris-EDTA buffer (pH 9) for 15 min. After cooling at room temperature during 20 min the endogenous peroxidase was inactivated and tissues incubated with primary monoclonal mouse anti-human IL-26 antibody at a dilution of 1/100 or mouse IgG2b isotype control during 30 min. Bound antibody was detected using the Envision FLEX-kit, code K8012 (Dako, Copenhagen Denmark). Slides were then rinsed in distilled water, counterstained in Hematoxylin, rinsed in running tap water, dehydrated, cleared, and mounted. The average area of the sections was 1–2 mm^2^, and the whole tissue section was analyzed by IHC. The intensity of IL-26 protein level was evaluated semiquantitatively as negative (0), very weak ((+)), weak (+), moderate (++), or strong (+++) in different types of pulmonary cells. These semiquantitative assessments were then assigned numerical values whereby the very weak ((+)) was given 1, the weak (+) 2, the moderate (++) 3, and the strong (+++) 4. Notably, the extent of the immunohistochemical expression levels for IL-26 was not separately assigned due to the positivity in all epithelial cells, i.e. percentage of the positive cells was 100.

#### mRNA analyses in unsorted BAL samples by hybridization

The mRNA was extracted from unsorted BAL cells (randomly selected smokers with (*n*=13) and without COPD (*n*=23) and healthy nonsmokers (*n*=16) according to the manufacturer’s instructions (NucleoSpin® miRNA kit, Macherey–Nagel, Düren, Germany). The mRNA integrity was assessed using the Bioanalyzer and thereafter amplified using the Low Input Quick Amplification Kit (Agilent Technologies) according to the manufacturer’s protocol, and subsequent Cy3-CTP labeling was performed by using one-color labeling kits (Agilent Technologies). Clean-up of the labeled and amplified probe was performed (Zymo Research Corporation, Irvine, CA). The size, distribution, and quantity of the amplified products were assessed by Nanodrop. Equal amounts of Cy3-labeled target were hybridized to Agilent human whole-genome 4x44K Inkjet arrays containing a total of 41000 probes corresponding to 19596 *entrez* genes. Hybridizations were performed at 65°C for 17 h at a rotation of 10 rpm. Arrays were scanned by using the Agilent microarray G2565BA scanner (Agilent Technologies) with Scan region: Agilent HD (61 × 21.6) and a resolution of 5 μm, TIFF: 16 bit, XDR: 0.10. Raw signal intensities (reported as log_2_ signal intensity) were extracted with Feature Extraction v10.1 software (Agilent Technologies). Notably, any gene with an average (log_2_ signal intensity) per group of less than 5.5 was below the limit of detection and was not considered. Flagged outliers were not included in any subsequent analyses.

#### Isolation, culture, and stimulation of AM

We ensured that the BAL samples (‘CYREBAC cohort’) did not contain red blood cells before they were used for cultures. In brief, 3 × 10^5^ unsorted BAL cells from healthy subjects were plated in a 24-well plate during 2 h in RPMI-1640 medium with l-glutamine (Fisher Scientific™), supplemented with FBS (10%) (Thermo Fisher Scientific, Uppsala, Sweden), and penicillin-streptomycin (1%) (Sigma-Aldrich™, Stockholm, Sweden AB). The nonadherent cells were then washed off (3×) with RPMI-1640 medium and the adherent cells starved overnight in fresh RPMI-1640 medium (supplemented with penicillin-streptomycin). The starvation medium was then replaced with another fresh RPMI-1640 medium (supplemented with penicillin-streptomycin) and the enriched AM were stimulated with vehicle or WTC (1%) during 24 h. Cell-free conditioned medium was harvested and IL-26 protein concentrations measured using ELISA. Notably, cytotoxicity/cell viability assays for this batch of prepared WTC was determined using AlamarBlue® Cell Viability Reagent assay on primary bronchial epithelial cells.

#### Analyses of mRNA in enriched AM by real-time PCR

Total RNA was isolated according to manufacturer’s protocol (Qiagen AB, Sweden) and the quality determined by Nanodrop. One microgram RNA was used to synthesize cDNA and RT-PCR was performed as previously described [8,12]. The primers used were according to our previous studies [8,12] (see Supplementary Table S5 for the primer sequences). Data were normalized (controls were set to 1) with reference to the housekeeping gene (*β-actin*) according to the *C*_T_ method (ΔΔ*C*_T_) and presented as fold differences for the actual mRNA, and reported as gene expression.

#### Preparation of WTC

The WTC were prepared as previously described [29,30]. In brief, mainstream smoke from a commercial cigarette (tar: 10 mg, nicotine: 0.8 mg, and CO: 10 mg/cigarette) (Marlboro™, Philip Morris®, Neuchâtel, Switzerland) was bubbled through 15 ml RPMI-1640 medium at room temperature using vacuum. Each cigarette was smoked during 4 min for a total of ten cigarettes. The solution was then filtered and frozen down as WTC at −80°C. The endotoxin concentration in the WTC has previously been proven undetectable [30].

#### Measurements of purulence, IL-8, and leukotriene B_4_ concentrations plus myeloperoxidase activity in IS samples

The IS purulence was determined using a colorimetric scale as previously described [31]. IL-8 was measured using Quatikine (R&D systems, Abingdon, U.K.), and leukotriene B_4_ (LTB_4_) using EIA from Cayman Chemical (Ann Arbor, MI, U.S.A.). as previously described [32]. Myeloperoxidase (MPO) activity was measured as previously described [22], according to the method of Axelsson et al. [33]. In brief, 3,3′,5,5′-TMB and hydrogen peroxide (H_2_O_2_) were added to the IS to measure MPO activity. The reaction was stopped with 2 M H_2_SO_4_ after 3 min, and analyzed at 450 nm. Horseradish peroxidase was utilized as standard.

#### Bacterial culture of BW and IS samples

Aliquots of BW and IS samples were sent for routine bacterial culture to the Department of Clinical Microbiology, Karolinska University Hospital and Department of Clinical Microbiology, Lund University Hospital, respectively and analyzed according to clinical routines [34]. In brief, the BW was cultured on blood, blood with Gentian Violet, cysteine lactose electrolyte deficient (CLED) and hematin agar plates. Blood and CLED agars were incubated under aerobic conditions, while blood with Gentian Violet and hematin agar were incubated in 5% CO_2_ at 36°C and evaluated after 24 and 48 h. Blood agar was also incubated under anaerobic conditions at 36°C and evaluated after 48 h. The *Streptococcus pneumoniae* was identified by optochin susceptibility, >13 mm; *Streptococcus pyogenes* (group A *Streptococcus*), agglutination for group A antigen (PhatoDxtra, Oxoid); *Staphylococcus aureus* with DNase and Staphaurex (Oxoid) detecting clumping factor and protein A. *Haemophilus influenzae* with XV test and *Pseudomonas aeruginosa* with colony morphology, oxidase, and API NE (BioMerieux) when appropriate ().

### Statistical analyses

Statistical analyses were performed using the GraphPad Prism® software, Inc, (San Diego, CA) and *P*-values <0.05 were considered statistically significant. Of note, *P*-values less than 0.001 were all presented as *P*<0.001. Nonparametric descriptive and analytical statistics were applied on nontransformed data unless otherwise stated. The Mann–Whitney, Wilcoxon signed ranked, Spearman’s rank correlation, Friedman tests, and linear regression analyses were utilized, where appropriate, to compute statistical differences. Notably, the Friedman’s test with Dunn’s multiple comparison post tests was used for trend analysis over time (stable clinical conditions, prior to exacerbation and during exacerbation). The parametric paired *t* test was used for the data from cells cultured *in vitro*.

## Results

### IL-26 protein in the airways of long-term smokers with or without COPD

**Figure 1:**
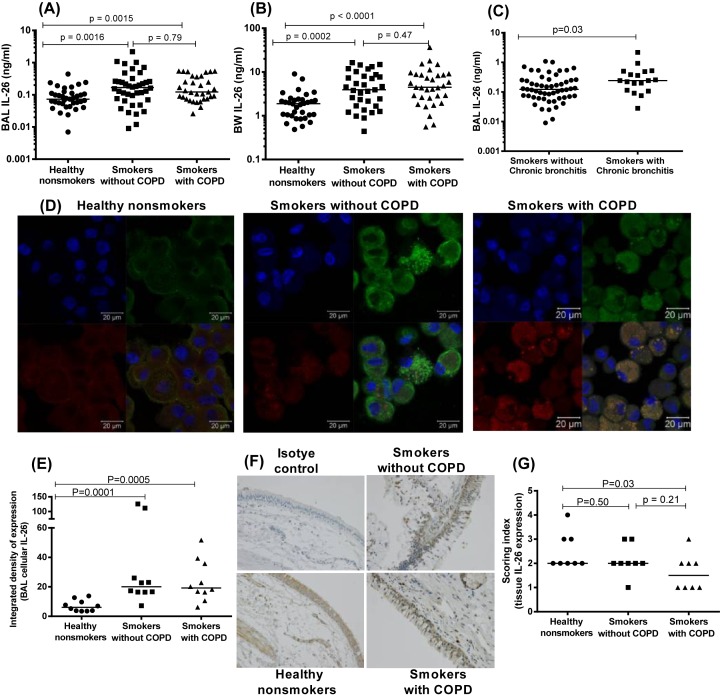
IL-26 protein in the airway lumen, airway leukocytes, and bronchial tissue biopsy samples from smokers and nonsmokers In this figure, datasets on human samples from one cohort (COSMIC cohort) are presented and all the IL-26 protein concentrations were quantitated in cell-free fluid samples using ELISA. (**A**) Concentrations of IL-26 in BAL fluid samples from healthy nonsmokers (*n*=37), smokers without COPD (*n*=40), and smokers with COPD (*n*=33). (**B**) Concentrations of IL-26 in BW fluid from healthy nonsmokers (*n*=34), smokers without COPD (*n*=33), and smokers with COPD (*n*=33). (**C**) Concentrations of protein in BAL fluid from smokers with or without COPD who have chronic bronchitis (*n*=18) and those without chronic bronchitis (*n*=55). (**D**) Representative pictures (ICF) for cellular IL-26 protein expression in AM from healthy nonsmokers (left panel), smokers without COPD (middle panel), and smokers with COPD (right panel). Each panel shows nuclear staining (blue) in the upper left quadrant, CD68 staining (of AM, red) in the lower left quadrant, IL-26 staining (green) in the upper right quadrant and CD68 and IL-26 co-staining (red and green) in the lower right quadrant. (**E**) The integrated density (CTCF using ImageJ after the ICF staining) of IL-26 expression in AM, *n*=8 for all groups. (**F**) Representative pictures (IHC) for the isotype control (upper left quadrant), IL-26 staining (brown) in healthy nonsmokers (lower left quadrant), IL-26 staining (brown) in smokers without COPD (upper right quadrant), and IL-26 staining (brown) in smokers with COPD (lower right quadrant). (**G**) The intensity of IL-26 expression in the bronchial tissue biopsies, *n*=8 for all groups. The horizontal lines in (A–C, E, G) indicate medians and the *P*-values are according to the Mann–Whitney test. The *P*-values <0.05 are considered significant. Concentrations of IL-26 on the *y*-axis (A–C) are represented in log scale.

To determine whether IL-26 is involved in the alteration of innate immunity in the airways of long-term smokers (referred to as ‘smokers’ from here and on), with or without COPD, airway samples including BAL, BW, and bronchial tissue biopsy samples from the COSMIC cohort were utilized. Healthy nonsmokers were included as controls (see Tables 1 and 2 for demographics). Extracellular concentrations of IL-26 protein were quantitated in the cell-free BAL and BW samples utilizing ELISA. We detected an increased average extracellular concentration of IL-26 in the BAL and BW samples from smokers, with or without COPD, compared with the healthy nonsmokers (Figure 1A,B and Supplementary Figure S1A,B). However, we found no statistically significant difference between the average extracellular concentration of IL-26 in the smokers with or without COPD (Figure 1A,B). In contrast, we found that in the pooled group of smokers with or without COPD, those with chronic bronchitis displayed a higher average extracellular concentration of IL-26 in BAL samples compared with those without chronic bronchitis (Figure 1C). Given that the group of smokers with COPD included both current and former smokers (‘ever-smokers’), we examined BAL and BW samples for these two groups but we found no clear differences in the average concentrations of IL-26 (Supplementary Figure S1C,D). The demographics for the current and former smokers in this cohort (COSMIC) are shown in Supplementary Tables S1 and S2. Given these similarities in IL-26 concentrations between smokers with and without COPD, as well as between current and former smokers with COPD, we henceforth refer to all of them as ‘smokers with or without COPD’.

We also characterized cellular IL-26 protein levels in BAL cells and bronchial tissue biopsy samples using ICF and IHC, respectively. Here, we found an increased average level of cellular IL-26 protein in the BAL cells of smokers with or without COPD (Figure 1D,E and Supplementary Figure S1E). We also detected cellular IL-26 protein in bronchial tissue biopsies of smokers, with or without COPD (Figure 1F). Interestingly, in bronchial tissue biopsies from the smokers with COPD, the average level of cellular IL-26 protein was decreased, compared with the corresponding samples from healthy nonsmokers. However, there was no reproducible difference between the smokers with and smokers without COPD (Figure 1G). Moreover, in this respect, we found no clear differences between the healthy nonsmokers and the combined group of smokers with or without COPD (Supplementary Figure S1F).

### Expression of the *IL-26* gene and functionally related genes in the airways of smokers with and without COPD

**Figure 2:**
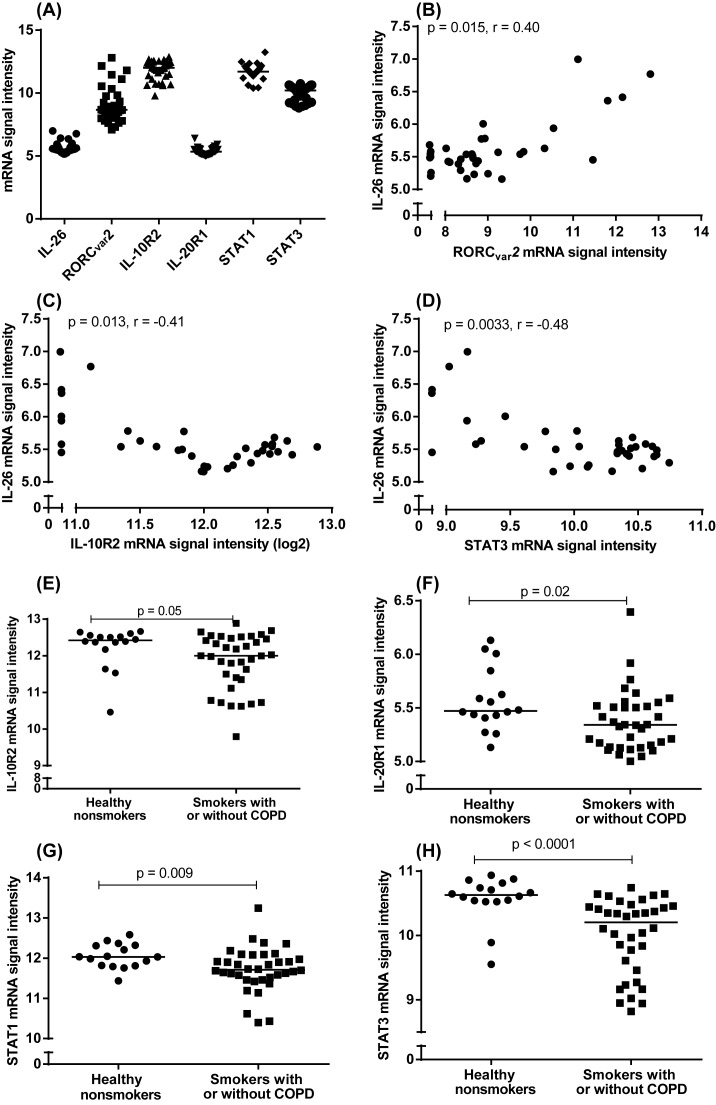
Expression of the *IL-26* gene and functionally related genes in airway cells from smokers with or without COPD In this figure, datasets on human samples from one cohort (COSMIC cohort) are presented and the mRNA expression was measured by hybridization. (**A**) mRNA signal intensities for inherent *IL-26, RORC_VAR2_, IL-10R2, IL-20R1, STAT1*, and *STAT3* genes in unsorted BAL cells from smokers with or without COPD (*n*=36). (**B**–**D**) Correlation of mRNA signal intensities of IL-26 and RORC_VAR2_ (B), IL-26 and *IL-10R2* (C) and *IL-26* and *STAT3* genes (D). (**E**–**H**) mRNA signal intensities of inherent *IL-10R2* (E), *IL-20R1* (F), *STAT1* (G), and *STAT3* genes (H), in BAL cells from smokers with or without COPD (*n*=36) compared with healthy nonsmokers (*n*=16). The horizontal lines in (E–H) indicate medians. The data in (B–D) as well as the *P*-values indicted are according to the Spearman’s correlation test and the data in (E–H) are according to the Mann–Whitney test. The *P*-values <0.05 are considered significant.

The expression of the *IL-26* gene is associated with that of its functionally related genes IL-10R2, IL-20R1, signal transducer and activator of transcription (STAT) 1 (STAT1) and STAT3 in BAL cells and bronchial epithelial cells of healthy nonsmokers [8–10,12]. To determine whether the IL-26 and the functionally related genes, including the Th17 cell-programming transcription factor RAR related orphan receptor ((ROR)C_VAR2_) are expressed in smokers with or without COPD and how these expressions relate to one another, we performed gene expression analyses in unsorted BAL cells from the COSMIC cohort using microarray. Examining the genes of interest from this pool, we correlated the *IL-26* gene with the archetype Th17 cell-programming transcription factor RORC_VAR2_, the IL-26’s receptor subunits IL-10R2 and IL-20R2, as well as the IL-26 receptor signaling molecules STAT1 and STAT3. Here, we found that BAL cells from smokers with or without COPD indeed expressed these genes, as well as the *IL-26* gene (Figure 2A). We detected a modest positive correlation between the expression of *IL-26* and *RORC_VAR2_* genes (Figure 2B) and a modest negative correlation between the expression of IL-26 and IL-10R2 (Figure 2C) as well as between that of IL-26 and STAT3 (Figure 2D). When we compared expression levels for the smokers with or without COPD with those of healthy nonsmokers, we found that the referred group of smokers expressed the lowest average level of *IL-10R2* (Figure 2E), *IL-20R1* (Figure 2F), *STAT1* (Figure 2G), and *STAT3* (Figure 2H) genes. Notably, IL-10R2 displayed only a trend toward a similar difference.

### IL-26 production and release in AM in response to WTC

**Figure 3:**
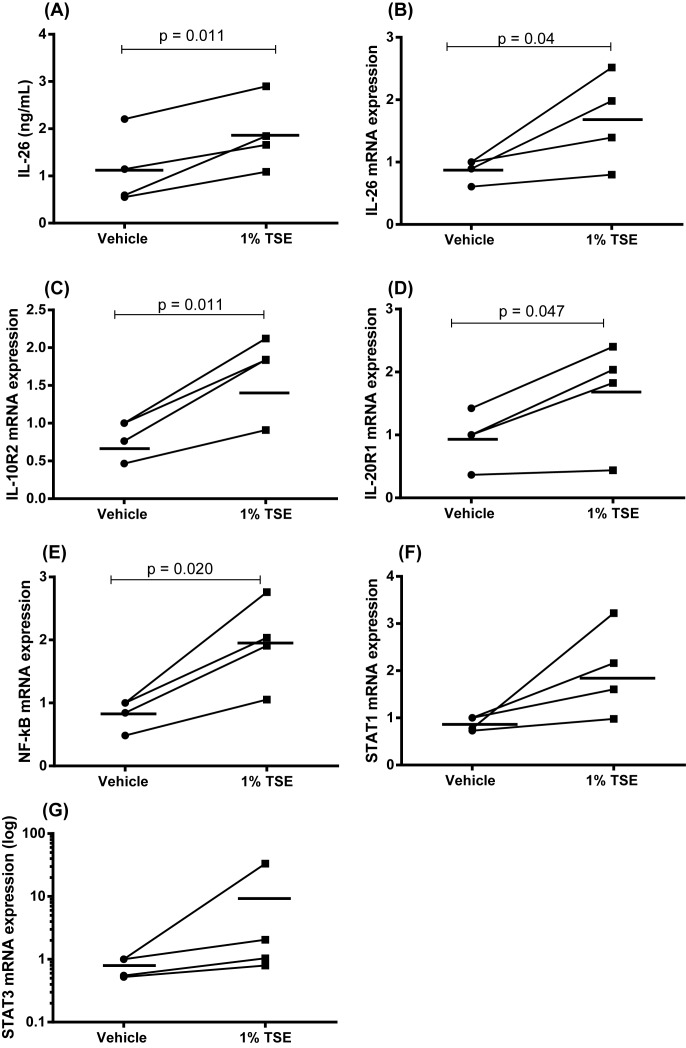
IL-26 production in AM and gene expression of functionally related genes in response to WTC *in vitro* In this figure, datasets on human samples from one cohort (CYREBAC cohort) are presented. The AM were enriched from BAL cell samples harvested from healthy volunteers and stimulated with WTC. The concentrations of IL-26 in the cell-free conditioned media were measured using ELISA. Gene expression analyses on the cells were performed using RT-PCR. (**A**) IL-26 protein concentrations in cell-free conditioned media from AM (*n*=4). The rest of the graphs show gene expression for (**B**) *IL-26*, (**C**) *IL-10R2*, (**D**) *IL-20R1*, (**E**) *NF-κB*, (**F**) *STAT1*, and (**G**) *STAT3* in AM. The *P-*values are according to the parametric paired *t* test whereby (A,B) are according to the one-tailed test and (C–G) according to the two-tailed test. The horizontal lines indicate means and the *P*-values <0.05 are considered significant.

To address a potentially important cellular source of IL-26 in the tobacco smokers, we examined whether stimulation of AM with WTC causes gene expression or protein release of IL-26 *in vitro*. In addition, we examined the gene expression for the IL-26 receptor complex (*IL-10R2 and IL-20R1*), the downstream intracellular signaling molecules (*STAT1 and STAT3*) and the transcription factor *nuclear factor κB (NF-κB)* in the AM. Thus, AM were enriched from BAL samples harvested from healthy nonsmokers in the CYREBAC cohort [8] and stimulated with WTC during culture (24 h) *in vitro*. In this setting, WTC caused a clear increase in extracellular IL-26 protein in the conditioned medium (Figure 3A) as well as of the expression of the *IL-26* gene (Figure 3B). Here, WTC also increased gene expression of *IL-10R2* (Figure 3C), *IL-20R1* (Figure 3D), and *NF-κB* (Figure 3E). However, WTC caused only a trend toward a positive response for the gene expression of *STAT1* and *STAT3* in AM (Figure 3F,G).

### IL-26 protein in the airways of smokers with COPD during stable clinical conditions and during exacerbations

**Figure 4:**
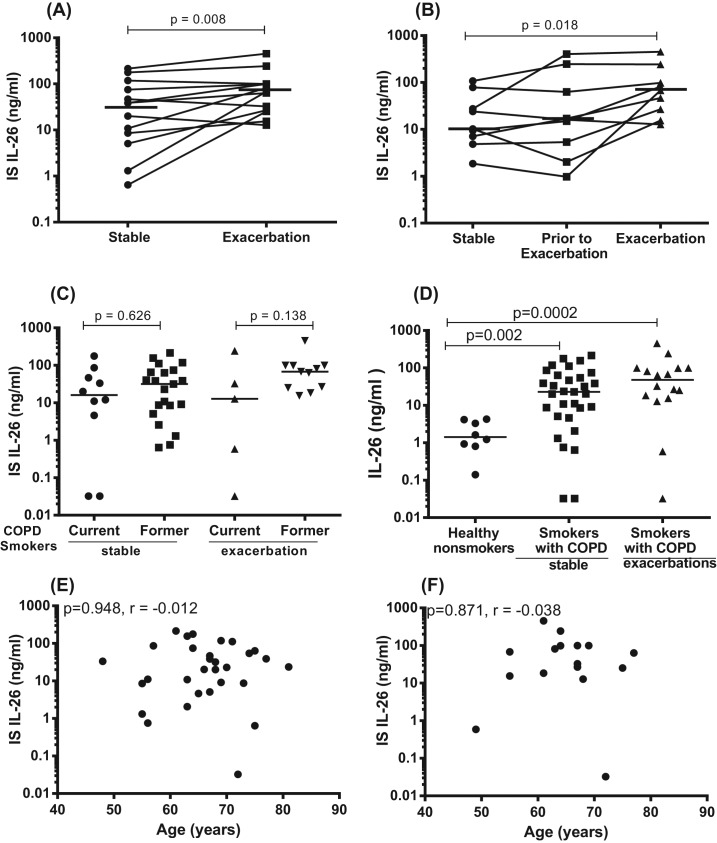
IL-26 protein concentrations in IS of smokers with COPD, in relation to exacerbations and age In this figure, datasets on human samples from one cohort (BALO cohort) are presented and all the IL-26 protein concentrations were quantitated in cell-free IS fluid samples using ELISA. (**A**) Concentrations of IL-26 in IS fluid during stable clinical conditions compared with exacerbations (*n*=13), (**B**) concentrations of IL-26 in IS fluid over time; during stable clinical conditions, prior to exacerbation and during exacerbation (*n*=9). (**C**) Concentrations of IL-26 in IS fluid from COPD-current smokers (*n*=10) and COPD-former smokers (*n*=21) during stable clinical conditions and for COPD-current smokers (*n*=5) and COPD-former smokers (*n*=11) during exacerbations. (**D**) Concentrations of IL-26 in IS fluid from healthy nonsmokers without COPD (*n*=8) and smokers with COPD (*n*=31) and COPD exacerbations, as assessed during stable clinical conditions (*n*=16). (**E**) Concentrations of IL-26 in IS fluid samples from smokers with COPD in relation to age during stable clinical conditions (*n*=31). (**F**) Concentrations of IL-26 in IS fluid samples from smokers with COPD, in relation to age during exacerbations (*n*=16). The *P-*values are according to Wilcoxon signed ranked test for (Figure 3A), Friedman test (Figure 3B), Mann–Whitney test (Figure 3C,D), and Spearman’s rank correlation (Figure 3E,F). The *P-*values <0.05 are considered significant. Concentrations of IL-26 on the *y*-axis are represented in log scale. Notably, the term “stable” in the figure panels signifies “stable clinical conditions” in the legend.

Given the clinical impact of COPD exacerbations [1,2,35], we determined whether local IL-26 is altered during these exacerbations in smokers with COPD. Repeated IS sampling was thus performed in smokers with COPD over time, during stable clinical conditions and during exacerbations (see Table 3 for demographics). Utilizing ELISA, we found that the average extracellular concentration of IL-26 in matched IS samples was increased during exacerbations in comparison with the corresponding concentration during stable clinical conditions (Figure 4A). For a subgroup of these patients, it was possible to identify an intermediary time-related category (pre-exacerbation), representing the most recent time point preceding an exacerbation and occurring (median (range)) 17 (14–23) days prior to the clinical event. When performing this analysis, we detected a modest increase in the average concentration of extracellular IL-26 protein over time; a time-dependent increase that proved to be statistically significant even though certain patients displayed aberrant trends (Figure 4B). Furthermore, we examined but found no pronounced differences in the average IL-26 concentration in IS samples harvested in current and former smokers with COPD (Figure 4C). The demographics for the current and former smokers in this cohort are shown in Supplementary Table S3. We also quantitated extracellular concentrations of IL-26 in IS from healthy nonsmokers and found that the average IL-26 concentration was much higher in samples harvested during stable clinical conditions in the smokers (current plus former smokers) with COPD than in the healthy nonsmokers, and this was even more true for the samples harvested during exacerbations (Figure 4D). Here, it should be pointed out that, in this case, the healthy nonsmokers tended to be younger (23 (21–39) years) than the smokers with COPD (65 (48–81) years). However, the data from the BALO cohort indicated no correlation between extracellular IL-26 protein concentrations in IS samples and age, regardless whether these were harvested during stable clinical conditions (Figure 4E) or during exacerbation (Figure 4F). In analogy with these findings, our data on IL-26 protein concentrations in BAL and BW samples from smokers with or without COPD from the COSMIC cohort during stable clinical conditions did not indicate any statistically significant correlation with age either (Supplementary Figure S2A,B).

### Correlation of IL-26 protein in the airways with smoking history and lung function

**Figure 5:**
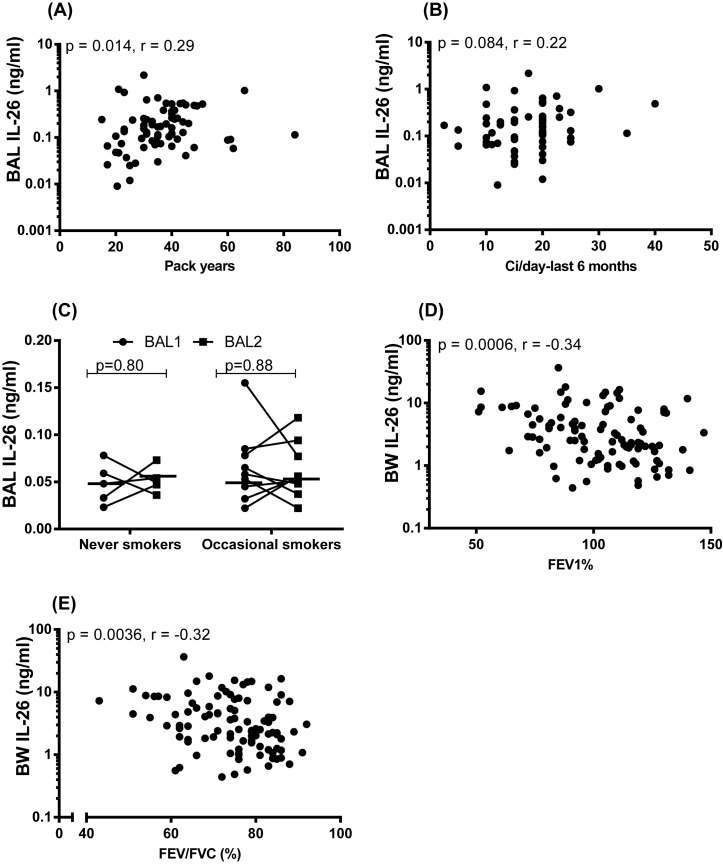
IL-26 protein concentration in relation to long-term and short-term exposure to tobacco smoke and lung function In this figure, datasets on human samples from three cohorts (COSMIC, Smoke Expo, and CYREBAC cohort) are presented and all IL-26 protein concentrations were quantitated in cell-free fluid samples using ELISA. (**A**) Concentrations of IL-26 in BAL fluid samples from smokers with or without COPD (COSMIC cohort), in relation to pack years (*n*=73). (**B**) Concentrations of IL-26 in BAL fluid samples from smokers with or without COPD (COSMIC cohort) in relation to the number of cigarettes smoked per day within the last 6 months (*n*=63). (**C**) Concentrations of IL-26 in BAL fluid samples from occasional smokers without COPD after smoking ten cigarettes within 48 h (*n*=9) and in never-smokers without COPD (*n*=5) (Smoke Expo cohort). (**D**) Concentrations of IL-26 in BW fluid samples (COSMIC cohort) in relation to FEV_1_% predicted (**E**) and FEV_1_/FVC% (*n*=100). The *P*-values are according to Spearman’s rank correlation test (A,B,D,E), Mann–Whitney U test (C). The horizontal lines in (C) indicate medians. The *P*-values <0.05 are considered significant. Concentrations of IL-26 on the *y*-axis (A,B,D,E) are represented in log scale. Abbreviations: FEV_1_, forced expiratory volume in 1 s; FVC, forced vital capacity.

We determined whether tobacco load relates to the increased extracellular concentration of IL-26 in the airways of smokers. We did this for historic (pack-years) and current (number of cigarettes smoked *per* day within the last 6 months) tobacco load by relating it to the extracellular concentration of IL-26 protein in BAL and BW samples of smokers with or without COPD from the COSMIC cohort and in the IS of smokers with COPD during stable conditions and during COPD exacerbations from the BALO cohort. In doing so, we observed a modest positive correlation between the extracellular concentration of IL-26 in BAL samples and historic tobacco load (Figure 5A) whereas this was not the case for current tobacco load (Figure 5B). For the extracellular concentration of IL-26 in the BW and IS samples, we observed no corresponding correlations (Supplementary Figure S3A–F).

We also examined whether short-term exposure to tobacco smoke alters extracellular IL-26 concentrations locally in the airways of healthy occasional smokers (without COPD) *in vivo* by utilizing samples from the Smoke Expo cohort (see Supplementary Table S4 for demographics). For this purpose, we analyzed BAL samples that were harvested during two bronchoscopies, performed 2 weeks before and 48 h after occasional smokers did smoke ten filtered cigarettes [25]. Examining the extracellular concentration of IL-26 protein in these BAL samples, we found no pronounced differences before and after the short-term exposure to tobacco smoke and we controlled for repeated bronchoscopy by examining never-smokers who refrained from smoking but still were investigated with the same protocol for repeated bronchoscopy (Figure 5C).

Moreover, we determined whether the increased extracellular concentration of IL-26 protein is associated with alterations in lung function assessed with spirometry in the COSMIC and BALO cohorts. To do this, we examined the respective correlation between, on the one hand, extracellular concentrations of IL-26 in BAL, BW, and IS samples from smokers with or without COPD and, on the other hand, forced expiratory volume (FEV)_1_% predicted and FEV_1_/forced vital capacity (FVC) (FEV%), collected during stable clinical conditions for each subject. However, we were unable to detect any correlation between IL-26 concentrations and these measures of lung function in the BAL and BW samples (Supplementary Figure S4A–D) or in the IS samples during stable clinical conditions (Supplementary Figure S4E,F) or during exacerbations (Supplementary Figure S4G,H). In contrast, when the healthy nonsmokers were included in our respective analysis for the BW and BAL samples, we did detect a modest and statistically significant negative correlation between IL-26 concentrations in BW samples from the COSMIC cohort, and FEV_1_% predicted (Figure 5D) on the one hand, as well as FEV% (FEV_1_/FVC) (Figure 5E), on the other hand. However, we were unable to identify a corresponding correlation for the concentrations of IL-26 in BAL samples (COSMIC cohort, see Supplementary Figure S4I,J).

### Correlation of IL-26 protein with colonization by pathogenic bacteria in airway samples

**Figure 6:**
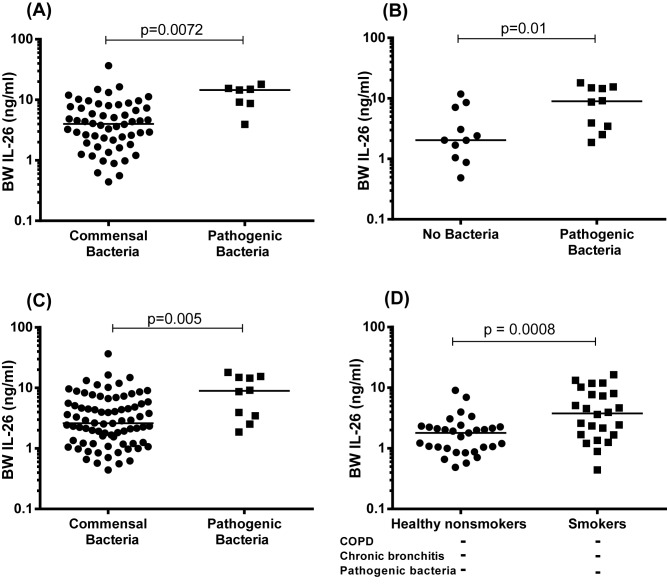
IL-26 protein concentrations in BW samples in relation to growth of pathogenic bacteria In this figure, datasets on human samples from one cohort (COSMIC cohort) are presented and all IL-26 protein concentrations in the cell-free BW fluid were quantitated using ELISA. (**A**) Concentrations of IL-26 in relation to growth of pathogenic (*n*=7) compared with commensal (*n*=54) bacterial species in samples from smokers with or without COPD. (**B**) Concentrations of IL-26 in relation to growth pathogenic bacterial species (*n*=10) compared with no growth of bacterial species (*n*=11) in samples from smokers with or without COPD, plus healthy nonsmokers. (**C**) Concentrations of IL-26 in relation to growth of pathogenic (*n*=10) compared with commensal (*n*=78) bacterial species in samples from smokers with or without COPD plus healthy nonsmokers. (**D**) Concentrations of IL-26 in samples from smokers without COPD, without chronic bronchitis and without growth of pathogenic bacterial species (*n*=24), compared with samples from healthy nonsmokers with no growth of pathogenic bacterial species (*n*=31). The horizontal lines indicate medians and the p-values are according to the Mann-Whitney test. The p-values < 0.05 are considered significant. Concentrations of IL-26 in the y-axis are represented in log scale.

We have previously shown that intrabronchial exposure to the bacterial compound endotoxin induces a clear increase in extracellular IL-26 protein concentrations in healthy nonsmokers *in vivo* [8]*.* Given this previous observation, we here examined how the increase in these extracellular IL-26 concentrations in smokers with or without COPD relates to the colonization by pathogenic bacteria. Thus, we performed culture of bacteria for BW samples from smokers with or without COPD during stable clinical conditions from the COSMIC cohort, as well as for IS samples from smokers with COPD during stable clinical conditions and during exacerbations from the BALO cohort. The detected bacteria are presented in Supplementary Table S6. In the BW samples from smokers with or without COPD that displayed growth of at least one species of pathogenic bacteria, there was a clearly higher average concentration of IL-26 protein compared with those displaying growth of commensal bacteria (here, synonymous to oral flora) (Figure 6A). Moreover, when we included the respective group of healthy nonsmokers in the analysis, we also observed that the average extracellular concentration of IL-26 was clearly higher in subjects that had growth of pathogenic bacteria compared with those without any growth of bacteria in the matching samples (Figure 6B). This was true for the whole population of subjects (healthy nonsmokers plus smokers with or without COPD) when comparing those with growth of pathogenic bacteria to those with growth of commensal bacteria (Figure 6C). Now, given the detected association of IL-26 with growth of pathogenic bacteria, and with chronic bronchitis, we subsequently excluded the particular subjects with these conditions amongst the smokers with or without COPD in the COSMIC cohort. Thus, in a separate analysis assessing the specific impact of long-term tobacco smoking on the increased concentrations of IL-26 in BW samples, we then found that the smokers still displayed increased average extracellular concentration of IL-26 protein in the referred samples, compared with the healthy nonsmokers in the same cohort (Figure 6D).

**Figure 7:**
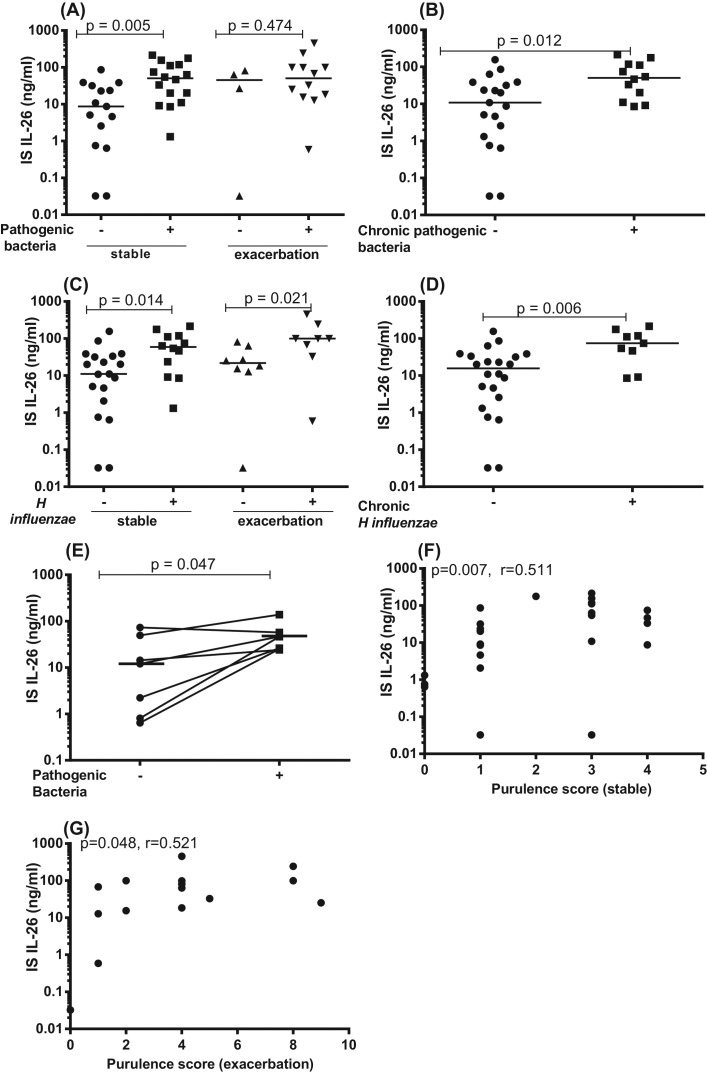
IL-26 protein concentrations in IS samples in relation to growth of pathogenic bacteria In this figure, datasets on human samples from one cohort (BALO cohort) are presented. The IL-26 protein concentrations were quantitated in cell-free fluid samples using ELISA, and bacterial growth in IS fluid samples from smokers with COPD were determined. (**A**) Concentrations of IL-26 in relation to growth of pathogenic bacterial species (*n*=16) compared with no growth of pathogenic bacterial species (*n*=15) during stable clinical conditions, as well as in relation to growth of pathogenic bacterial species (*n*=12) compared with no growth of pathogenic bacterial species (*n*=4) during exacerbations. (**B**) Concentrations of IL-26 in relation to chronic growth of pathogenic bacterial species (*n*=12) compared with no chronic growth of pathogenic bacterial species (*n*=19) during stable clinical conditions. (**C**) Concentrations of IL-26 in relation to growth of *H. influenzae* (*n*=11) compared with no growth of *H. influenzae* growth) (*n*=20) at any visit during stable clinical conditions, as well as in relation to growth of *H. influenzae* (*n*=8) compared with no growth of *H. influenzae* growth (*n*=8) during exacerbations. (**D**) Concentrations of IL-26 in relation to chronic growth of *H. influenzae* (+) (*n*=9) compared with no chronic growth of *H. influenzae* (–) (*n*=22) during stable clinical conditions. (**E**) Concentrations of IL-26 within the same subjects when there is growth of pathogenic bacterial species and when there is no growth of pathogenic bacterial species (*n*=7)during stable clinical conditions. (**F**) Correlation between IL-26 concentrations and purulence score during stable clinical conditions (*n*=31) and (**G**) during exacerbations (*n*=16). The horizontal lines indicate medians and the *P*-values are according to Mann–Whitney test (Figure 6A–H), Wilcoxon signed ranked test (Figure 6I), Spearman’s rank correlation (Figure 6J,K). The *P*-values <0.05 are considered significant. Concentrations of IL-26 on the *y*-axis are represented in log scale. Notably, the term “stable” in the figure panels signifies “stable clinical conditions” in the legend.

When analyzing the data from bacterial cultures of IS samples from smokers with COPD in the BALO cohort, we found that the subjects with COPD that had growth of pathogenic bacteria in IS samples at any time point during stable clinical conditions did consistently display a clearly increased average extracellular concentration of IL-26 in the very same IS samples, in comparison with the subjects who did not display this growth at any time point (Figure 7A). However, this was not the case for these subjects during exacerbations of COPD, as we detected no corresponding difference in the concentrations of IL-26 in relation to the detection of bacteria (Figure 7A). Furthermore, we observed that the smokers with COPD who consistently had growth of at least one species of pathogenic bacteria in the IS samples at all visits during stable clinical conditions (chronic growth of pathogenic bacteria) did display an increased average IL-26 concentration, compared with the subjects who did not (Figure 7B). Here we were unable to perform a matching analysis of the corresponding conditions during exacerbation, due to a too small sample size (i.e. only one subject displayed repeated exacerbations). Given that the majority of the subjects with growth of pathogenic bacteria displayed *H. influenzae* in their IS samples, we examined these subjects separately. We then found that the subjects having growth of *H. influenzae* at any time point displayed increased average extracellular concentrations of IL-26 in their IS samples, both during stable clinical conditions and during exacerbations, in comparison with those who did not display this specific growth of *H. influenzae* at any time point (Figure 7C). In addition, subjects who had growth of *H. influenzae* at all visits during stable clinical conditions (chronic growth of *H. influenzae*) did display increased extracellular concentrations of the IL-26 protein, compared with subjects who did not (Figure 7D). Here, just like above, we were unable to perform a matching analysis of the corresponding conditions during exacerbation, due to a too small sample size (i.e. only one subject displayed repeated exacerbations). To further characterize the association between IL-26 and growth of pathogenic bacteria in IS samples, we performed paired analyses where we compared IL-26 concentrations within the same subject(s), when they displayed growth of pathogenic bacteria or no growth during stable clinical conditions. Amongst the qualifying smokers with COPD, we then found that the average IL-26 concentration in IS was indeed increased when the bacteria were detected in comparison with when they were not detected (Figure 7E). However, this comparison was not possible during exacerbations given that only one subject experienced an exacerbation during more than one occasion (one visit) during the course of the study. Finally, we also quantitated the purulence of IS samples, a validated indicator of bacterial presence in the airways [22,36]. We then found that the extracellular concentrations of IL-26 in IS samples correlated with purulence in a positive manner, both during stable clinical conditions (Figure 7F) and during exacerbations (Figure 7G) amongst the referred smokers with COPD.

### Correlation of IL-26 protein with markers of neutrophil accumulation in airway samples

**Figure 8:**
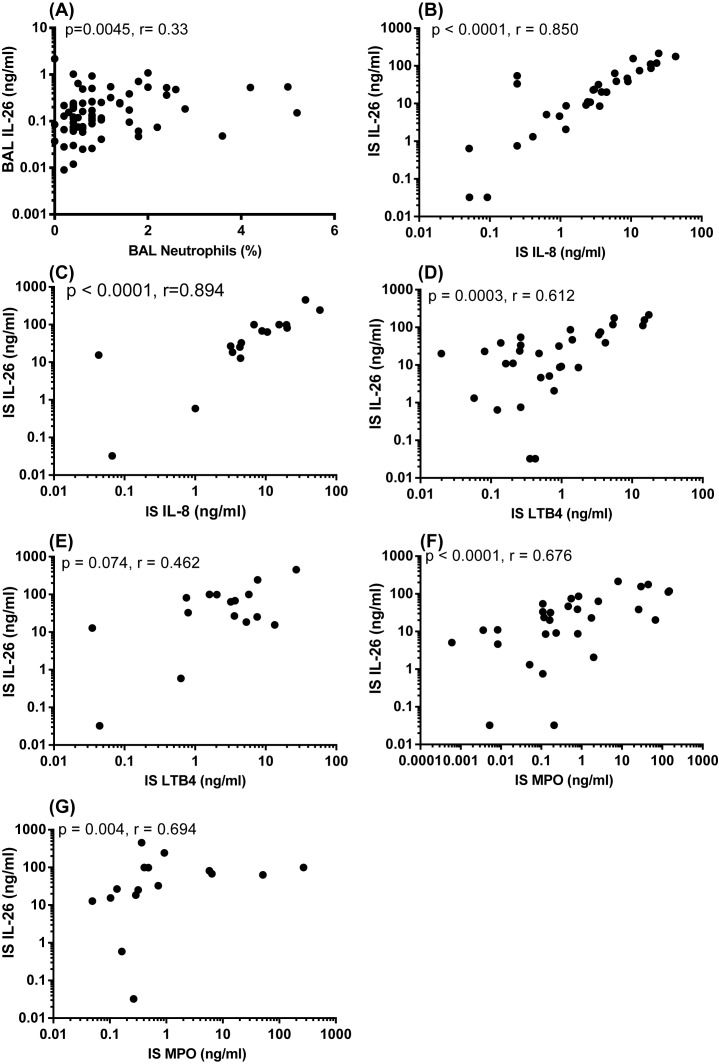
Correlation of IL-26 concentrations with markers of neutrophil accumulation in IS samples In this figure, datasets from two human cohorts (COSMIC and BALO cohorts) are presented and IL-26 protein concentrations in the cell-free fluid samples were quantitated using ELISA. (**A**) Correlation between concentrations of IL-26 and neutrophils in BAL samples from smokers with or without COPD (COSMIC cohort: *n*=73). (**B**) Correlation between concentrations of IL-26 and IL-8 (ELISA) during stable clinical conditions (*n*=31) and (**C**) during exacerbations (*n*=16) in IS fluid samples from smokers with COPD (BALO cohort). (**D**) Correlation between concentrations of IL-26 and LTB_4_ (ELISA) during stable clinical conditions *(n*=31) and (**E**) during exacerbations (*n*=16) in IS fluid samples from smokers with COPD (BALO cohort). (**F**) Correlation between concentrations of IL-26 and MPO activity (ELISA) during stable clinical conditions (*n*=31) and (**G**) during exacerbations (*n*=16) in IS fluid samples from smokers with COPD (BALO cohort). The data and *P-*values indicated are according to the Spearman’s correlation test. The *P-*values <0.05 are considered significant. Concentrations of IL-26 on the *y*-axis are represented in log scale.

Concentrations of IL-26 and neutrophils in BAL samples are known to correlate in a positive manner for healthy nonsmokers *in vivo* [8]. Given this observation, we here examined how the extracellular concentrations of IL-26 in BAL samples from smokers with or without COPD in the COSMIC cohort relate to the corresponding neutrophil concentrations. We then observed a modest positive correlation between the concentrations of IL-26 and the percentage of BAL neutrophils (Figure 8A). In analogy, we examined the corresponding association of IL-26 concentrations with those of the neutrophil chemoattractants IL-8 and LTB_4_, as well as with the neutrophil activity marker MPO in IS samples from smokers with COPD in the BALO cohort. We then observed that these particular concentrations of IL-26 displayed a very strong and positive correlation with IL-8 during stable clinical conditions (Figure 8B), as well as during exacerbations (Figure 8C). Moreover, in the same matched samples, the concentrations of IL-26 and LTB_4_ displayed a clear and positive correlation during stable clinical conditions (Figure 8D) but not during exacerbations (Figure 8E). Finally, we found that the concentrations of IL-26 and MPO in the IS samples correlated in a strong and positive manner during stable clinical conditions (Figure 8F) as well as during exacerbations (Figure 8G).

### Effects of recombinant human IL-26 protein on the expression of pro-inflammatory genes in enriched AM

**Figure 9:**
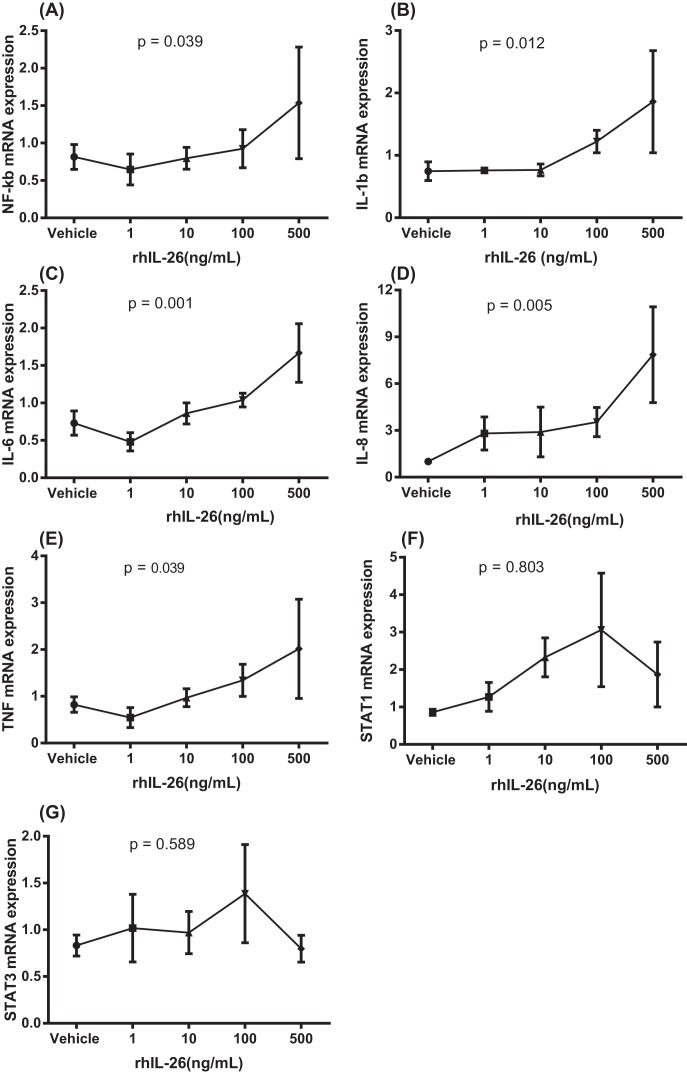
Effects of rhIL-26 on pro-inflammatory genes in human AM In this figure, datasets on human samples from one cohort (CYREBAC cohort) are presented. AM were enriched from BAL cells harvested during bronchoscopy in healthy volunteers and these AM were stimulated with different concentrations of rhIL-26. Gene expression analyses were performed using RT-PCR. Graphs show gene expression for (**A**) *NF-κB* (*n*=3), (**B**) *IL-1β* (*n*=4), (**C**) *IL-6* (*n*=4), (**D**) IL-8 (*n*=4), (**E**) *TNF-α* (*n*=4), (**F**) *STAT1* (*n*=4), and (**G**) *STAT3* (*n*=4). Data are presented as mean and S.E.M., *P*-values are according to the linear regression analyses and the *P*-values <0.05 are considered significant.

We examined the effects of recombinant human (rh) IL-26 on the expression of *NF-κB, IL-1β, IL-6, IL-8*, and *TNF-α* genes as well as genes for the IL-26 receptor complex (*IL-10R2 and IL-20R1*) and the intracellular signaling molecules (*STAT1 and STAT3*) in the AM cultured *in vitro*. Here, the AM were stimulated with different concentrations of rhIL-26 protein during 24 h. This stimulation caused a concentration-dependent increase in gene expression for *NF-κB* (Figure 9A), *IL-1β* (Figure 9B), *IL-6* (Figure 9C), *IL-8* (Figure 9D), and *TNF-α* (Figure 9E). However, rhIL-26 caused no corresponding statistically significant effect on the gene expression of *STAT1* (Figure 9F) or *STAT3* (Figure 9G).

## Discussion

In the present study on IL-26 in the airways of long-term tobacco smokers, we made four fundamental observations. First, we observed that the smokers, with or without COPD, displayed an increased average extracellular concentration of IL-26 protein in BAL, BW, and IS samples, in comparison with the corresponding samples from healthy nonsmokers. However, we were unable to prove that the smokers with COPD differ from those without COPD during stable clinical conditions in this respect. Second, we observed that smokers with COPD displayed an enhanced average extracellular concentration of IL-26 protein during exacerbations, in comparison with stable clinical conditions in the very same subjects. Third, we observed that smokers with or without COPD, who also have chronic bronchitis, displayed an increased average concentration of IL-26 in BAL samples, in comparison with those who did not have chronic bronchitis. Fourth, smokers with or without COPD, who had growth of pathogenic bacteria in BW and IS samples, also had an increased average concentration of IL-26 in the matching airway samples, in comparison with those who did not have growth of pathogenic bacteria in the corresponding samples. Thus, our original observations argue that local extracellular IL-26 protein is increased by long-term smoking *per se*, with additional enhancement during exacerbations of COPD, in chronic bronchitis and during colonization by pathogenic bacteria.

The idea that long-term tobacco smoking *per se* increases extracellular concentrations of IL-26 in the airways was addressed in several ways in the present study. To start with, we analyzed BW samples from smokers lacking COPD, chronic bronchitis, and growth of pathogenic bacteria, and we found that these smokers still exhibited a higher average concentration of IL-26 compared with the healthy nonsmokers. For further scrutiny, we utilized two experimental models, one *in vivo* and one *in vitro*, to evaluate the impact of short-term exposure to tobacco smoke components on IL-26. In the human *in vivo* model, we found no sustained difference in the average extracellular concentration of IL-26 in human BAL samples harvested 2 weeks before and within 48 h after the short-term consumption of ten cigarettes. However, we did detect two types of signals in the human *in vitro* model. Here, after the 24 h exposure of AM to WTC *in vitro*, we observed both a reproducible enhancement in the expression of the *IL-26* gene in the cells and an increase in the extracellular concentrations of IL-26 protein in the conditioned media. Taken together, these findings can be interpreted as an argument for a transient effect of short-term exposure to tobacco smoke on the production of IL-26 in human airways. However, given our results in the experimental *in vivo* model, it appears feasible that the integrative immune regulation in healthy human airways *in vivo* is capable of rapidly normalizing this transient response to tobacco smoke components.

Interestingly, we found that stimulation of the AM with the WTC also increased expression of the genes for the IL-26 receptor complex (*IL-10R2 and IL-20R1*) and for the generic, pro-inflammatory transcription factor *NF-κB*. Notably, the simultaneous release of IL-26 protein and expression of the IL-26 receptor genes induced by WTC in AM argue that there is a positive feedback stimulation of the AM by the IL-26 protein. This is particularly true given that stimulation of AM with rhIL-26 enhanced the expression of the genes for the neutrophil-mobilizing cytokines *IL-1β, IL-6, IL-8*, and *TNF-α*, as well as the transcription factor *NF-κB*. Here, it is noteworthy that the involvement of NF-κB in the release of IL-26 in bronchial epithelial cells has been demonstrated [8], which means that the transcription factor NF-κB may be involved in both the production of and the response to IL-26 in AM.

We observed no pronounced differences in the average extracellular concentrations of IL-26 in BAL, BW, and IS samples of current and former smokers with COPD. Just like the results from our experimental models on short-term exposure to tobacco smoke components, this can be interpreted as if the increased local concentration of IL-26 in smokers is a consequence of chronic inflammation due to long-term tobacco smoking and thus, not a mere result of current smoking *per se*. This interpretation is also supported by the positive but modest correlation between historic tobacco load and local extracellular concentrations of IL-26 in the BAL samples and the lack of a corresponding correlation with current tobacco load. The seemingly contradictory lack of verified correlations between, on the one hand, IL-26 concentrations in BW or IS samples and, on the other hand, historic tobacco load may be explained in at least two ways. First, the BW and IS samples are less likely to reflect conditions in the peripheral airways than the BAL samples. Second, it is possible that the BW and IS sampling are less reproducible than BAL sampling and requires a larger sample size to achieve a comparable statistical power. Thus, taken together, our current findings are all compatible with chronic inflammation due to long-term tobacco smoking being required for establishing the enhanced local concentrations of the neutrophil-mobilizing cytokine IL-26 that signifies smokers with or without COPD. Moreover, our current findings suggest that, once triggered, the pathogenic release of IL-26 may continue even after smoking cessation.

Although our datasets indicate that local IL-26 is increased in long-term smokers regardless of whether they have COPD or not, notwithstanding, these datasets also indicate that IL-26 is involved in the course of COPD. Specifically, we found that the average extracellular concentration of IL-26 in IS samples from smokers with COPD was enhanced during exacerbations, compared with stable clinical conditions in the same subjects. Moreover, we found that by average, the extracellular concentration of IL-26 protein in the IS samples displayed a modest but time-dependent increase prior to COPD exacerbations, a potentially important finding given the clinical need for biomarkers predicting upcoming COPD exacerbations [2,37]. Of note here, the average time point for the critical sampling ahead of the actual exacerbation occurred as much as 17 days ahead of the clinical event, something that motivates future analysis of time points closer to the clinical event. In addition to this, we found that the extracellular concentrations of IL-26 in BW samples from smokers with or without COPD did correlate in a negative manner with FEV_1_ (% pred) and FEV %, the two critical lung function parameters utilized for the diagnoses and monitoring of the disease severity in COPD patients. The detection of a statistically significant correlation between concentrations of IL-26 in BW samples compared with lung function and the lack of a matching correlation for concentrations of IL-26 in BAL samples may relate to what level of the airways these samples actually represent from an anatomical point-of-view [38,39]. The BW samples which represent the large airways are likely to represent conditions in a more relevant location from a pathophysiological point-of-view, where the main part of the airway obstruction is likely to occur, than is the case for BAL samples representing the smaller airways. Moreover, the herein utilized cohort (COSMIC), included smokers with relatively modest airway obstruction and this fact may have made it more critical to utilize the type of samples that are harvested really close to the relevant site in terms of pathogenic events. Finally, we found that bronchial tissue biopsies from smokers with COPD expressed lower levels of IL-26 protein compared with nonsmokers whereas this was not the case when we compared smokers without COPD to the same nonsmokers. This observation *per se* provides evidence for a weaker immune response in the tissue of smokers with COPD compared with that of smokers without COPD. It is also noteworthy that, in contrast with the decreased levels of IL-26 in the tissue biopsies, containing an abundance of bronchial epithelial cells from smokers with COPD, we found increased levels of IL-26 in the AM from the bronchoalveolar space compared with the nonsmokers. This observation *per se* provides evidence for a stronger immune response in the bronchoalveolar space in the smokers with COPD compared with the nonsmokers. Taken together, these four findings are all compatible with IL-26 being involved in the course of COPD.

Chronic bronchitis is an important morbidity amongst long-term smokers, independently of COPD [26,40], and chronic bronchitis also represents a severe comorbidity in COPD [40–42]. In our current study, we observed that smokers with or without COPD who have chronic bronchitis display an increased average extracellular concentration of IL-26 in BAL samples. We think that this association might be of importance for the determination of risk for disease progression in long-term smokers, with or without COPD. This association thereby merits further investigation.

We also found increased extracellular concentrations of IL-26 in BW and IS samples from smokers, with or without COPD, who displayed growth of pathogenic bacteria during stable clinical conditions in the same samples. Given that increased susceptibility to bacterial infections is also a morbidity amongst long-term smokers, with or without COPD [3,6,35,43], our finding supports the idea that local IL-26 is involved in the alterations of innate immunity in these subjects. It is true that it remains to be persued whether this link to bacterial colonization is a cause or a consequence. Therefore, it is important to recall that, although the growth of pathogenic bacteria, chronic bronchitis as well as exacerbations of COPD are associated with an increase in local IL-26, this does not undermine the conclusion that long-term tobacco smoking *per se* does enhance IL-26 in the airways.

We observed that IS samples from smokers with COPD display a substantially increased average extracellular concentration of IL-26 protein compared with healthy nonsmokers constituting controls. A mechanistically interesting observation in the referred smokers was that the IL-26 concentrations in the IS samples correlated with neutrophil-mobilizing mediators in the same samples. Thus, there was a strong, positive correlation between IL-26 and IL-8 and a strong correlation with LTB_4_ during stable clinical conditions only. However, we found a matching positive trend during exacerbations and the lack of a statistically significant correlation with LTB_4_ during exacerbation may relate to less of statistical power given a smaller *n* and, most likely, larger biological variability during exacerbations. Notably, there was also a corresponding, positive correlation with the neutrophil activity marker MPO during both clinical conditions. In addition, we detected a positive correlation between the concentrations of extracellular IL-26 and neutrophils in BAL samples from long-term smokers with or without COPD, even though this particular correlation was relatively weak. Tentatively, our findings are compatible with IL-26 playing an active role in the local accumulation of neutrophils in the airways of tobacco smokers with or without COPD [8–10,41,42,44].

Our assessment of mRNA in the airways *in vivo* focussed on the expression of the *IL-26* gene in relation to several functionally associated genes in unsorted BAL cells from smokers with or without COPD (COSMIC cohort) as well as in AM from healthy nonsmokers (CYREBAC cohort)*.* We observed that the smokers with or without COPD, displayed decreased expression of genes for *IL-10R2, IL-20R1, STAT1*, and *STAT3* compared with their corresponding controls. On the contrary, when we examined the same molecules after stimulation of AM with WTC *in vitro*, we found that this experimental stimulus increased the expression of *IL-10R2* and *IL-20R1* genes; there was a similar positive trend for the *STAT1* and *-3* genes. We find the rationale for this discrepancy uncertain. However, one explanation is that, during acute exposure of airways to tobacco smoke components, the AM may increase their expression of IL-26 and its receptor complex but during chronic exposure to tobacco smoke components, the AM may respond by down-regulating the expression of the IL-26 receptor complex as a means to curb the inflammation caused by IL-26. It is also noteworthy that the experimental models, cell compositions, duration of stimulations, and the exact contents of the stimuli differ for the unsorted BAL cell samples and the AM. Moreover, we observed that expression of the *IL-26* gene correlates with the archetype Th17 cell-programming transcription factor *RORC_VAR2_* gene in a positive manner in BAL cell samples harvested *in vivo* [45]. We found this latter finding to be compatible with IL-26 being functionally linked to Th17 cells, presumably due to the established fact that Th17 cells can produce IL-26 [14,46–48]. We also found that the *IL-26* gene correlates with the IL-26 receptor subunit *IL-10R2* in a negative manner, and this was also true for the intracellular signaling molecule *STAT3* gene, possibly due to a protective mechanism here as well. However, we did not observe any corresponding correlation with the constitutive gene expression of the receptor subunit *IL-20R1* nor with the gene expression of the intracellular signaling molecule *STAT1*. We speculate that the suggested type of protective mechanism(s) serves to limit long-term activation of innate neutrophil accumulation and thus, to limit chronic inflammation.

In conclusion, the findings of the present study indicate that local IL-26 protein in the airways is increased by long-term but not by short-term tobacco smoking *per se*. The findings of the present study also indicate that there is a further increase in local IL-26 in pulmonary morbidities amongst long-term tobacco smokers, such as chronic bronchitis and COPD exacerbations, as well as in subjects colonized by pathogenic bacteria. Thus, the local release of IL-26 may relate both to the inflammatory setting and to the presence of certain bacteria, implying true pathogenic relevance for morbidities in long-term tobacco smokers. The pathogenic relevance of local IL-26 is also supported by the previous documentation of its neutrophil-mobilizing effect and the current finding that its local concentration correlates with several signs of neutrophil accumulation, to high tobacco load and to poor lung function. Moreover, our experimental data from AM suggests that IL-26 does enhance the gene expression of classic pro-inflammatory cytokines. Tentatively, IL-26 emerges as a promising molecular target for improving the understanding of the pathogenic mechanisms behind clinically-important pulmonary morbidities in long-term tobacco smokers. Whether or not IL-26 in the airways may constitute a useful target for improved clinical diagnosis, monitoring or even therapy of pulmonary morbidities amongst long-term tobacco smokers does deserves further study.

## Clinical perspectives

- The immunological alterations in the airways of long-term smokers with or without COPD and chronic bronchitis are characterized by an excessive accumulation of neutrophils in parallel with an enhanced frequency of bacterial infections in the airways; the role of the neutrophil mobilizing cytokine IL-26 has not yet been investigated in this clinical context.
- Local IL-26 is increased in the airways of long-term smokers, with or without COPD, even more so in those with chronic bronchitis, local growth of pathogenic bacterial species and exacerbations of COPD, while also being associated with poor lung function, neutrophil mobilization, and pro-inflammatory cytokine signaling.
- The cytokine IL-26 emerges as a promising molecular target in the airways to monitor for improving the understanding of the pathogenic mechanisms behind pulmonary morbidities in tobacco smokers.

## Supporting information

**Supplementary Fig 1:** 

**Supplementary Fig 2:** 

**Supplementary Fig 3:** 

**Supplementary Fig 4:** 

## Abbreviations

AM: alveolar macrophage
BAL: bronchoalveolar lavage
BW: bronchial wash
CLED: cysteine lactose electrolyte deficient
COPD: chronic obstructive pulmonary disease
CT: threshold cycle
CY3-CTP: cytidine 3 - cytidine triphosphate
ELISA: enzyme-linked immunosorbent assay
FEV1: forced expiratory volume in 1 s
FVC: forced vital capacity
ICF: immunocytofluorescence
IHC: immunohistochemistry
IL: interleukin
IS: induced sputum
LABA: long-acting β agonist
LAMA: long-acting muscarinic antagonist
LTB_4_: leukotriene B_4_
MPO: myeloperoxidase
NF-κB: nuclear factor κB
rhIL-26: recombinant human IL-26
RORC_VAR2_: RAR related orphan receptor
RPMI: ross park memorial institute
SABA: short-acting β agonist
SAMA: short-acting muscarinic antagonist
STAT: signal transducer and activator of transcription
Th: T-helper
TNF: tumor necrosis factor
WTC: water-soluble tobacco smoke component
