# Supplementary figures and images for: The neutrophil-mobilizing cytokine interleukin-26 in the airways of long-term tobacco smokers
Source: Clin Sci (Lond). 2018 May 21;132(9):959–83. doi: 10.1042/CS20180057 (PMC6365630; doi:10.1042/CS20180057)

Supplementary Fig. 1

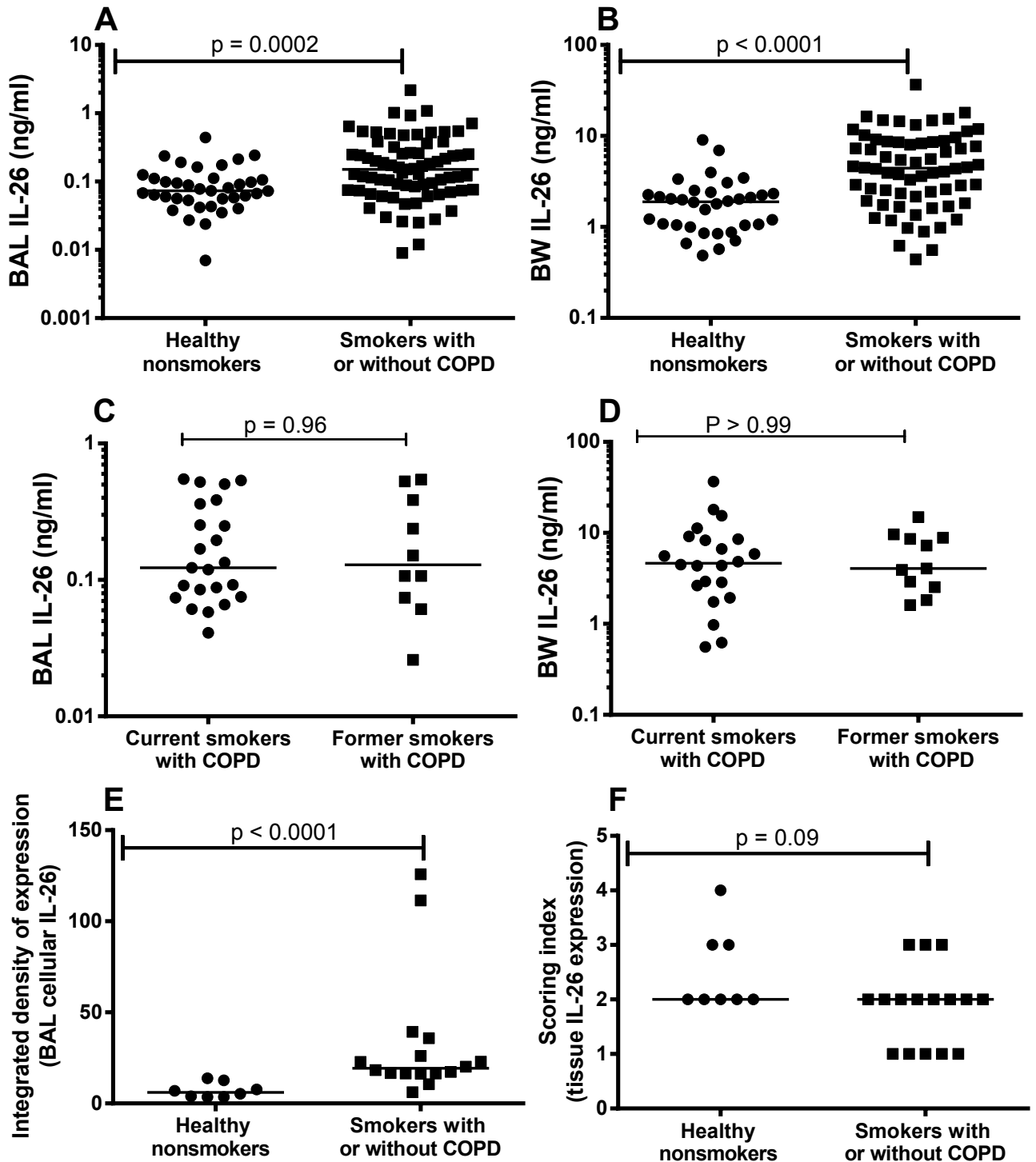

**Supplementary Fig. 2**

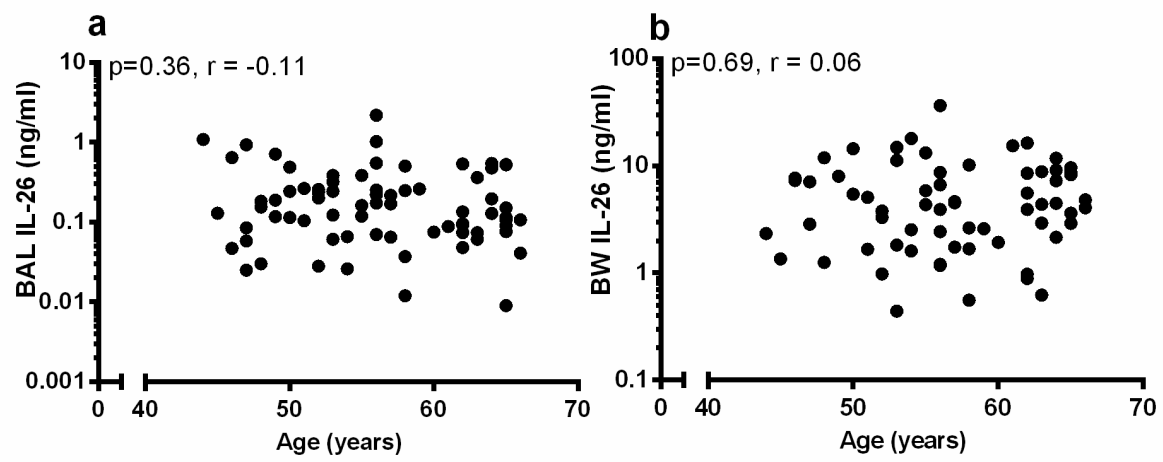

# Supplementary Fig. 3

**A**

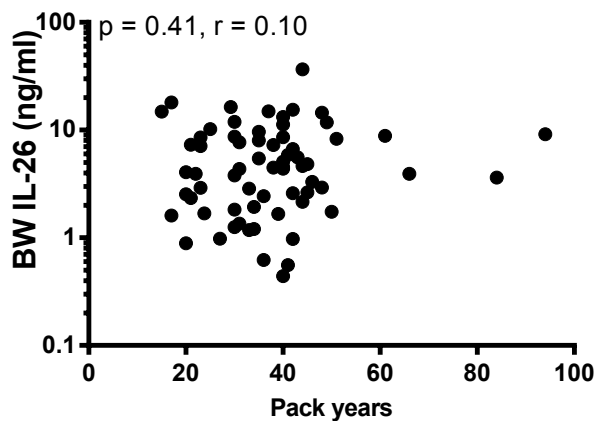

**B**

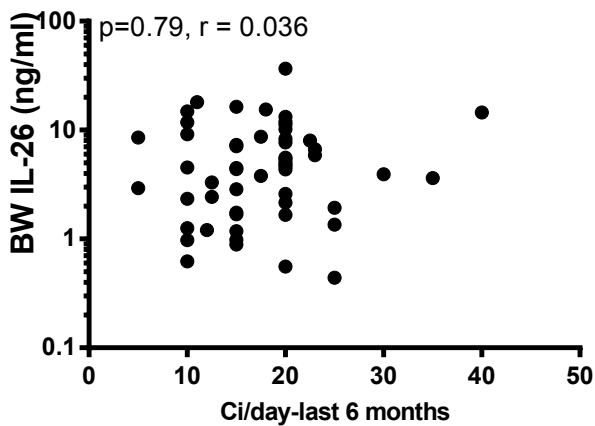

**C**

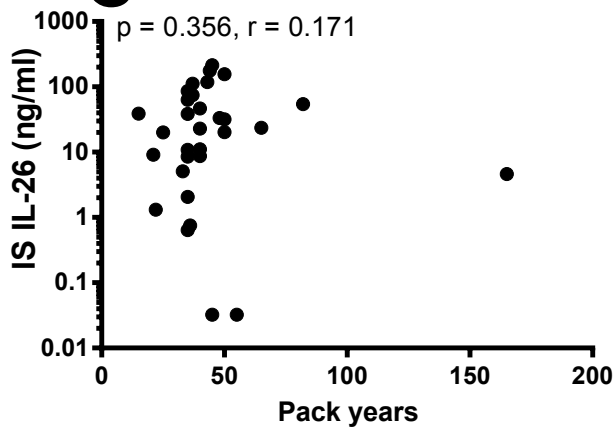

**D**

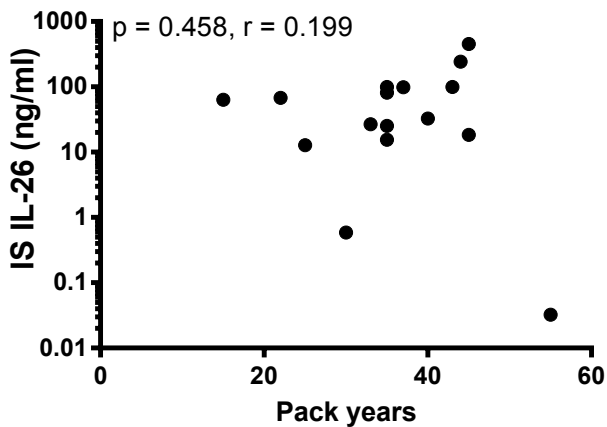

**E**

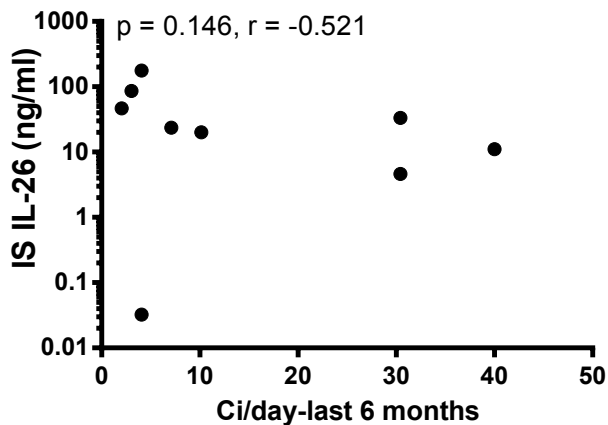

**F**

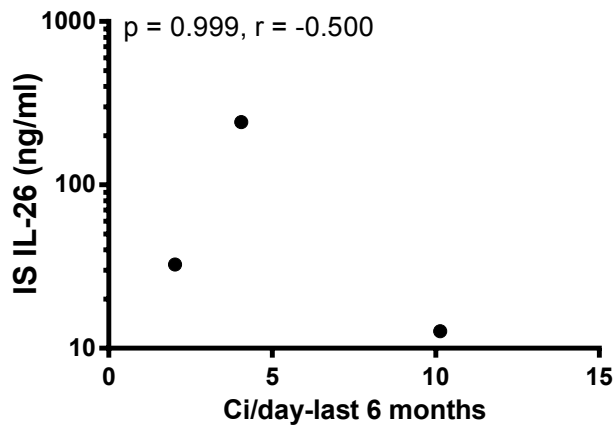

# Supplementary Fig. 4

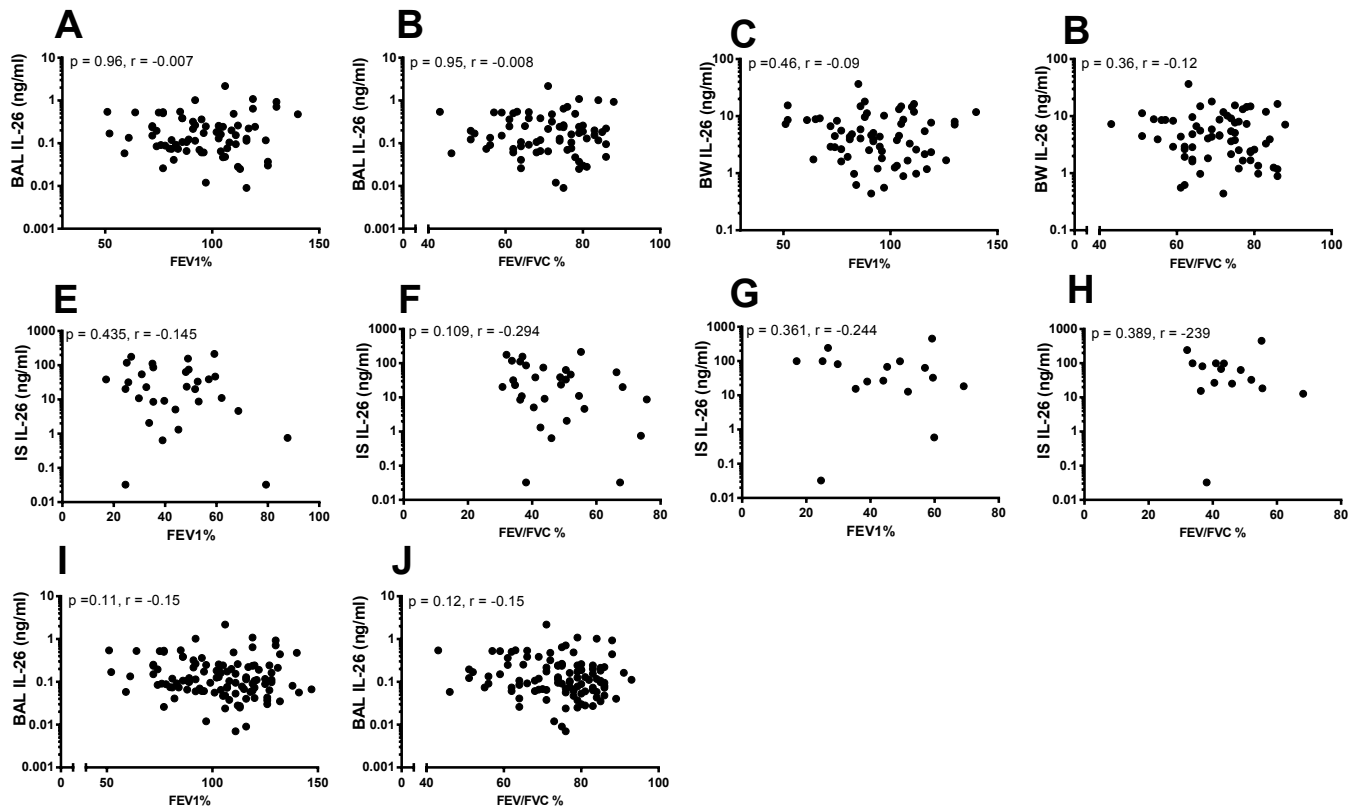

Supplement: Supplementary file 1 [file cs-132-cs20180057_supp1.pdf]
